# Association between secondhand smoke exposure in pregnant women and their socioeconomic status and its interaction with age: a cross-sectional study

**DOI:** 10.1186/s12884-022-04968-6

**Published:** 2022-09-09

**Authors:** Wensu Zhou, Xidi Zhu, Zhao Hu, Shaojie Li, Baohua Zheng, Yunhan Yu, Donghua Xie

**Affiliations:** 1 Department of Information Management, Maternal and Child Health Hospital of Hunan Province, Changsha, China; 2 Department of Social Medicine and Health Management, Xiangya School of Public Health, Central South University, Changsha, China; 3 Department of Epidemiology and Health Statistics, Xiangya School of Public Health, Central South University, Changsha, China

**Keywords:** Pregnancy, Socioeconomic status, Secondhand smoke, Cross-sectional study, China

## Abstract

**Background:**

Existing evidence highlights that exposure to secondhand smoke (SHS) is a risk factor for pregnant women’s health and is possibly affected by individual characteristics. This study aimed to explore the effect of individual socioeconomic status (SES) on SHS exposure among pregnant women in the third trimester and the interaction effect of age.

**Methods:**

A total of 678 nonsmoking pregnant women with a median age of 29.0 years from 14 communities in a medium-sized city were recruited for this survey. Exposure to SHS was defined as the self-reported smoking habit of a spouse/partner. Individual SES characteristics consisted of marital status, educational attainment, employment and per capita monthly income.

**Results:**

There were 238 (35.1%) participants who suffered from SHS exposure. Compared to the pregnant women who were employed, those who were unemployed were more likely to suffer from SHS exposure (OR = 1.572, 95% CI: 1.013–2.441). Participants who had a high school or technical secondary school education were more likely to be exposed to SHS than those with a college education or above (OR = 1.601, 95% CI: 1.029–2.449). Advanced age was a protective factor for participants with a college education or above (OR = 0.939, 95% CI: 0.884–0.997), but age increased the risk of SHS exposure among women who had unstable marriages (OR = 1.256, 95% CI: 1.019–1.549).

**Conclusion:**

Exposure to SHS was very common among pregnant women in the third trimester. Pregnant women with a low SES and an older age should be considered a key population for the implementation of public health interventions.

## Introduction

The most well-known unhealthy habit, smoking, is common among adults, especially males. Globally, nearly 50% of males are tobacco users; the smoking rate is higher in developing countries than in developed countries [1]. China has the highest tobacco production and the most tobacco consumers in the world, and approximately 47.2% of Chinese males had smoking habits in an investigation conducted in 2013 [2]. Thus, with an increasing number of smokers, many nonsmoking people, especially pregnant women, children and elderly individuals, are more likely to suffer from passive tobacco exposure (so-called secondhand smoke (SHS)) [3, 4]. Over the past few decades, many epidemiological studies have also suggested that the exposure of pregnant women to SHS by their partners was very common during pregnancy, even though they were nonsmokers [4–6]. Two cross-sectional studies conducted in Henan and Sichuan Provinces reported that approximately 60 to 70% of pregnant women experience SHS exposure, and 75.1% of nonsmoking pregnant women suffer from chronic SHS exposure from their spouses [6, 7]. Additionally, some investigations indicated that the self-reported rate of SHS exposure was relatively high in the third trimester [8–10]. Previous studies have shown that exposure to SHS is more serious than active smoking [3, 11, 12] because it is 3 to 4 times more damaging per gram of particulate matter than smoke directly inhaled by a smoker [13]. An increasing number of studies have indicated that exposure to SHS affects almost every type of adverse pregnancy outcome, such as depression disorders, low levels of health-related quality of life, preterm labor, rupture of membranes, and fetal weight loss [3, 14, 15]. Moreover, SHS includes at least 70 carcinogenic substances, increasing the risks of sudden infant death syndrome, cancers, and chronic kidney and respiratory diseases in children [16].

Socioeconomic status (SES), which is considered a construct, mainly involves many factors, such as educational attainment, occupational status, income and wealth [17]. SES reflects an individual’s social position relative to other members of society; it also indicates a person’s capacity for resources [18, 19]. An increasing number of studies have indicated that SES is associated with an individual’s health behaviors, attitudes and outcomes [20]. Previous studies reported that individual characteristics of SES not only affected active smoking behavior but were also linked with SHS exposure [21–23]. However, whether SES affects SHS exposure during pregnancy is debatable. For instance, Madureira et al. reported that a duration of educational attainment over 13 years was a protective factor for reducing environmental tobacco exposure during pregnancy [23], which was similar to other studies [4, 24, 25]. The probability of daily SHS exposure at home was negatively associated with pregnant women’s household income and family wealth [4, 24]. However, although researchers found that occupation was significantly associated with active smoking and SHS exposure during pregnancy [26, 27], Reece and colleagues did not identify this association [4]. Moreover, many researchers have found marital status to be consequential for health and considered it a positive indicator linked to reducing harmful exposure and engaging in health behavior [28], but statistical significance was not reported in two studies focused on the relationship between marital status and SHS exposure during pregnancy [29, 30]. In addition, the magnitude of SHS exposure and its association with SES could vary by region, as previous studies showed great heterogeneity in cultural background, tobacco use and attitudes across countries [6, 29], which means that the conclusions drawn from different countries may not reflect the same situation in other countries. Some conclusions are expected to be updated when sufficient evidence from new research conducted with other populations becomes available.

In addition, the age of pregnant women was also considered an indicator for predicting SHS exposure during pregnancy, and it was positively associated with SHS exposure [29, 31, 32]. The accumulation hypothesis showed that the level of SES-based health advantages progressively declined with age [33]. This might suggest an interaction of age with SES. Moreover, age is an important risk factor for pregnant women because the older a woman is, the higher the risk of death or injury for the fetus. Thus, the effect of age and SES on SHS exposure is of particular interest, but the present literature is not clear on the connection.

In summary, the current study relied on a community-based sample to reflect the association between individual SES and SHS exposure among pregnant women in the third trimester and explored the interaction effect between age and SES on SHS exposure. It is of great importance to facilitate the design and implementation of effective public health prevention programs and policies.

## Methods

### Study design and participants

**Fig. 1:**
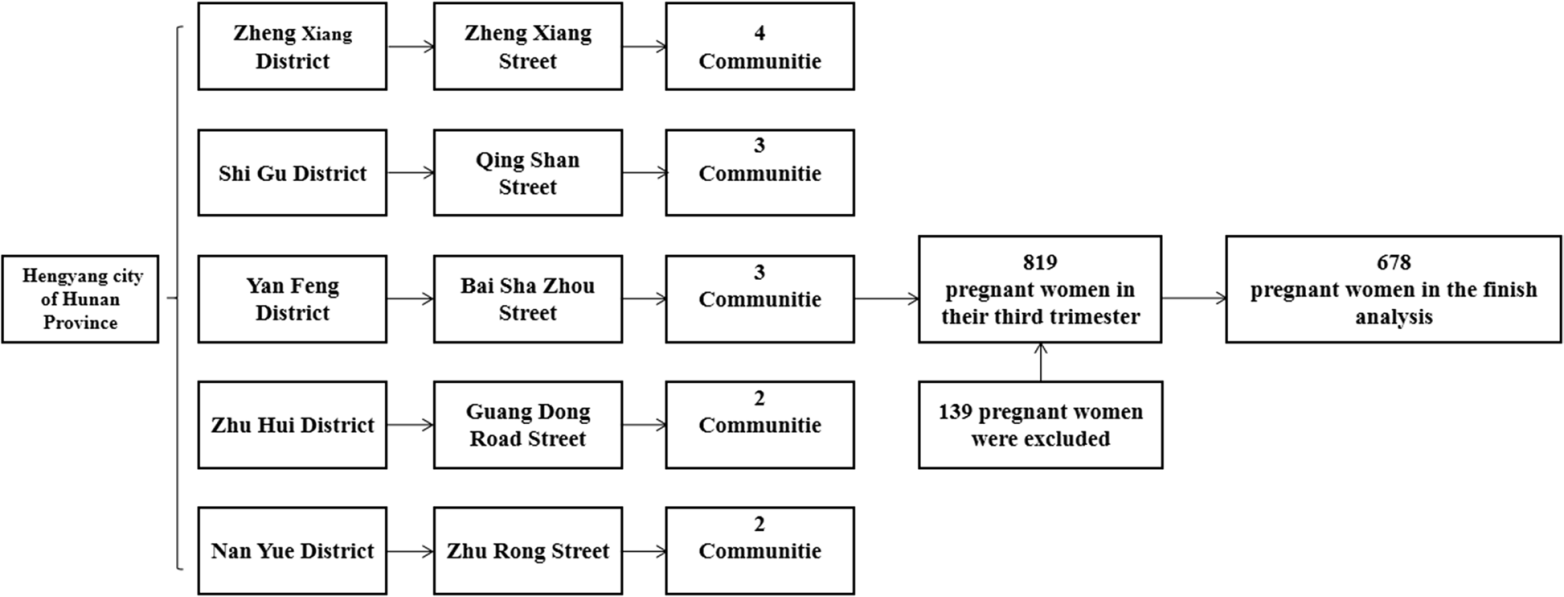
The selection flowchart of the current study

**Fig. 2:**
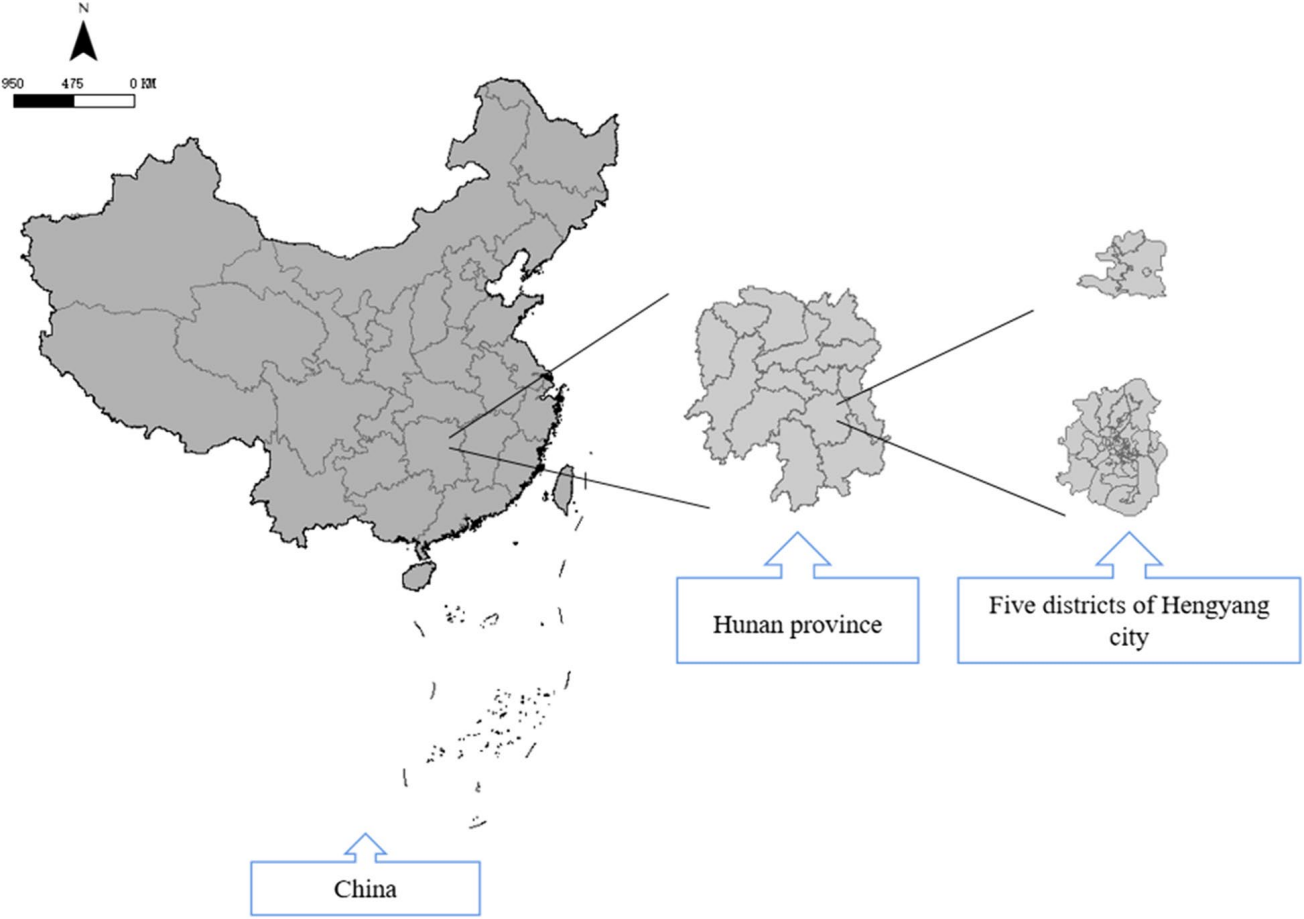
The geographic position of the five districts of Hengyang, Hunan Province, China

Data were derived from a cross-sectional study based on a community investigation conducted in Hengyang, Hunan Province, China, from July to September 2019. Hengyang is a typical industrial city located in Central China. The survey used a stratified random sampling strategy, with districts as the primary sampling unit. First, 5 streets were randomly selected from 5 districts of Hengyang. Then, communities were selected randomly based on communities and streets at a ratio of 3:1. Ultimately, 4 communities on Zhengxiang Street, 3 communities on Qingshan Street, 3 communities on Baishazhou Street, 2 communities on Guangdong Road Street and 2 communities on Zhurong Street with a total of 819 pregnant women in the third trimester were included in this study. The inclusion criteria were as follows: 1) women aged over 18 years; 2) women voluntarily participating in the project; 3) women with a registered pregnancy at a community health center and who had lived in the community for more than 6 months; 4) women who were nonsmokers during pregnancy; and 5) women living with their spouse/partner during pregnancy. Among the recruited women, 6 participants were excluded for missing information on smoking habits and exposure, and 135 participants were excluded due to not meeting the inclusion criteria. A total of 678 pregnant women in the 3rd trimester were included in the analysis. All participants were interviewed for approximately 20 mins and completed a structured questionnaire including relevant information about them. Moreover, the participants signed informed consent forms. The flow chart of this study is presented in Fig. 1, and Fig. 2 shows the geographic position of the five districts of Hengyang, Hunan Province, China.

### Ethical considerations

The study was carried out in accordance with the Declaration of Helsinki. Participants gave their informed consent to participate. This study was approved by the Ethics Committee of Xiangya School of Public Health of Central South University on 15 July 2019 (XYGW-2019-056).

### Assessment of SHS exposure

According to the definition for SHS in the Global Adult Tobacco Survey 2010 [34], all pregnant women were asked to respond to the question “Has your spouse/partner smoked at home at least 1 day per week while you have been pregnant?”. Those who responded “yes” and “always” were considered to be exposed to SHS at home during pregnancy. In particular, the definition we used in the study was a specific source of SHS because, for example, the women could be exposed at their workplace or at home by other inhabitants who smoked.

### Assessment of individual SES

Considering the characteristics of the status structure of society, in general, SES is defined as a multidimensional construct that jointly encompasses the assessment of the objective and subjective characteristics of a person [35, 36]. In the present study, we selected four items, including individual educational level, employment status, marital status and per capita monthly income, to measure individual SES according to previous studies [37, 38]. Among the items, educational level was categorized as junior middle school or below, high school or technical secondary school or college or above; employment status was categorized as yes or no; and marital status was classified as married and living together or divorced and living together/cohabiting relationship. In addition, per capita income per month was divided into three groups: ≤3000 RMB, 3001–7999 RMB and ≥ 8000 RMB.

### Covariates

Several variables that were included in the analysis were considered as covariates: age, ethnicity (Han and minority groups), and household registration status (hukou) (rural areas and urban areas). In particular, household registration status refers to China’s unique household registration (hukou) system, generally based on a person’s current/prior residential status [39]. The behavioral lifestyle of the participants included smoking habits (never or former), current exercising (yes or no) and current drinking (yes or no). Family-related factors included the educational attainment of spouses/partners (junior middle school or below, high school or technical secondary school or college or above), employment of spouses/partners (yes or no), drinking habits of spouses/partners in the last year (yes or no) and living with an aunt/uncle after getting married (yes or no). We also collected information on regular antenatal examinations (yes or no) and complications of pregnancy (yes or no).

### Statistical analysis

The characteristics of the participants are presented as the means and standard deviations for continuous variables that had a normal distribution, as the median and interquartile range (IQR) for continuous variables that did not have a normal distribution, or as numbers and percentages for categorical variables. The difference in exposure to SHS according to demographic characteristics, SES and family factors was examined using the chi-square test. The difference in age at SHS exposure was analyzed using a nonparametric Mann-Whitney U test since age was not confirmed to be normally distributed. Binary logistic regression analysis with the enter method was applied to explore the association between SES and exposure to SHS in the 3rd trimester among pregnant women after controlling for age, ethnicity, household registration status, smoking habit, current exercising and drinking, employment of spouses/partners, educational attainment of spouses/partners, drinking habits of spouses/partners in the last year, living with an aunt/uncle after getting married, regular antenatal examinations and complications of pregnancy. Model 1 was a multivariate logistic regression model that only included educational attainment, marital status, employment and per capita monthly income. Model 2 was a multivariate logistic regression model performed after adjusting for covariates. The following statistical tests were reported from the logistic regression models: the Hosmer–Lemeshow test, Cox & Snell R Square and Nagelkerke R Square. Finally, we fitted multivariate logistic regression models to test whether the association between age and SHS exposure differed by SES. We added cross-product terms (i.e., age *marital status or age *employment) into the regression models to examine the interaction effects and stratified the samples for estimation interpretation (Table 3) [40, 41]. Herein, marital status and employment were binary variables. For educational attainment and per capita monthly income, each variable had three categories, dummy variables were assigned to each variable, and we set the last category as a reference group [42]. Then, as shown in Table 4, the dependent and independent variables were set as SHS exposure and age, respectively. We conducted stratified analyses by SES to determine which SES group was more sensitive to SHS with an increase in age. We tested the statistical significance of differences between effect estimates of SES by calculating the 95% confidence interval (CI) as ${\overset{\frown }{Q}}_1-{\overset{\frown }{Q}}_2\pm 1.96\sqrt{{\left(S{\overset{\frown }{E}}_1\right)}^2+{\left(S{\overset{\frown }{E}}_2\right)}^2}$, where ${\overset{\frown }{Q}}_1$ and ${\overset{\frown }{Q}}_2$ are the estimates for the two categories, and $S{\overset{\frown }{E}}_1$ and $S{\overset{\frown }{E}}_2$ are their respective standard errors (Table 4).

Associations are presented as crude and adjusted odds ratios (ORs) with 95% confidence intervals (95% CIs). All analyses were conducted using R v.4.0.5. A *P value* < 0.05 was considered statistically significant.

## Results

### Demographics of the study sample

Table 1 shows the individual demographics and the comparison of the respondents. Overall, of the 678 pregnant women included in this community-based survey, 35.1% (238) had SHS exposure. The median age of the participants was 29.0 years (SD = 6.0). A total of 97.8% of the women were of Han ethnicity with an urban household registration status (73.2%). A total of 95.4% of the women were married and living with a partner/spouse and were employed (74.9%). The proportions of the participants who had a junior middle school education or below, high school or technical secondary school education and college education or above were 19.5, 23.7 and 56.8%, respectively. Regarding per capita income per month, 8.8, 71.1 and 20.1% of the women received less than 3000 RMB, between 3001 and 7999 RMB and more than 8000 RMB, respectively. Only 4.3 and 9.7% of the women reported that they had a smoking habit before pregnancy and currently drank. Regarding the characteristics of spouses/partners, 98.1% of the spouses/partners were employed, and 60.8% of them had a college education or above as well as drinking habits in the last year. A total of 64.0% of the participants lived with an aunt/uncle after getting married. The percentages of regular antenatal examinations and complications during pregnancy were 91.6 and 10.5%, respectively.

**Table 1:** Characteristics of the pregnant women in the 3rd trimester according to exposure to SHS n(%)/median (IQR)

Characteristic	Total *n* = 678	Never exposed to SHS *n* = 440	Current exposure to SHS *n* = 238	*Z/χ*^*2*^	*p* Value
**Demographic characteristic of the participants**					
Age	29.0 (6.0)	29.0 (6.0)	29.0 (6.0)	−0.748	0.455
Ethnicity					
Han	663 (97.8)	427 (64.4)	236 (35.6)	3.191	0.074
Minority group	15 (2.2)	13 (86.7)	2 (13.3)		
Household registration status (hukou)					
Rural area	182 (26.8)	115 (63.2)	67 (36.8)	0.319	0.572
Urban area	496 (73.2)	325 (65.5)	171 (34.5)		
Living with an aunt/uncle after getting married					
No	244 (36.0)	177 (39.4)	67 (27.5)	9.777	**0.002**
Yes	434 (64.0)	263 (60.6)	171 (39.4)		
Regular antenatal examinations					
Yes	621 (91.6)	392 (63.1)	229 (36.9)	10.190	**0.001**
No	57 (8.4)	48 (84.2)	9 (15.9)		
Complications of pregnancy					
No	607 (89.5)	391 (64.4)	216 (35.6)	0.590	0.442
Yes	71 (10.5)	49 (69.0)	22 (31.0)		
**Individual SES**					
Marital Status					
Married and living together	647 (95.4)	417 (64.5)	230 (35.5)	1.232	0.267
Divorced but living together/Cohabiting relationship	31 (4.6)	23 (74.2)	8 (25.8)		
Employment status					
Yes	508 (74.9)	346 (68.1)	162 (31.9)	9.184	**0.002**
No	170 (25.1)	94 (55.3)	76 (44.7)		
Educational attainment					
Junior middle school or below	132 (19.5)	82 (62.1)	50 (37.9)	4.091	0.129
High school or technical secondary school	161 (23.7)	96 (59.6)	65 (40.4)		
College or above	385 (56.8)	262 (68.1)	123 (31.9)		
Per capita monthly income in RMB					
≤ 3000	60 (8.8)	40 (66.7)	20 (33.3)	0.260	0.878
3001–7999	482 (71.1)	314 (65.1)	168 (34.9)		
≥ 8000	136 (20.1)	86 (63.2)	50 (36.8)		
**Behavioral lifestyle habits**					
Smoking habit					
Never	649 (95.7)	419 (64.6)	230 (35.4)	0.751	0.386
Former	29 (4.3)	21 (72.4)	8 (27.6)		
Currently exercising					
Yes	620 (91.4)	399 (64.4)	221 (35.6)	0.934	0.334
No	58 (8.6)	41 (70.7)	17 (29.3)		
Currently drinking					
No	612 (90.3)	408 (66.7)	204 (33.3)	8.645	**0.003**
Yes	66 (9.7)	32 (48.5)	34 (51.5)		
**Spouse/partner’s SES**					
Educational attainment of spouse/partner					
Junior middle school or below	94 (13.9)	66 (70.2)	28 (29.8)	3.051	0.218
High school or technical secondary school	172 (25.4)	117 (68.0)	55 (32.0)		
College or above	412 (60.7)	257 (64.2)	155 (37.6)		
Employment of spouse/partner					
Yes	665 (98.1)	431 (64.8)	234 (35.2)	0.001	0.970
No	13 (1.9)	9 (69.2)	4 (30.8)		
**Spouse/partner’s behavioral lifestyle habits**					
Drinking habit of spouse/partner in the last year					
No	252 (37.2)	119 (47.2)	133 (52.8)	54.998	**< 0.001**
Yes	426 (62.8)	321 (75.4)	105 (24.6)		

Values indicated in bold are statistically significant

After analyzing the differences under different demographic characteristic conditions, we observed the rate of SHS exposure was higher among participants who lived with aunt/uncle after getting married (39.4%) than others who were not (*χ*^*2*^ = 9.777, *p* = 0.002). The rate of SHS exposure was 36.9% among participants who regularly received antenatal examinations (*χ*^*2*^ = 10.190, *p* = 0.001), which was higher than others who were not. Unemployed participants were more likely to suffer from SHS exposure and the rate of SHS exposure was 44.7% (*χ*^*2*^ = 9.184, *p* = 0.002). The prevalence of SHS exposure was 51.5% in participants with drinking habits (*χ*^*2*^ = 8.645, *p* = 0.003). In contrast, we observed a lower proportion of SHS exposure in participants whose spouse/partner had drinking habits in the last year (24.6%) than counterparts (52.8%) unexpectedly (*χ*^*2*^ = 54.998, *p* < 0.001).

### The association between SES and SHS exposure among pregnant women in the 3rd trimester

We used binary logistic regression analysis to explore the association between SES and SHS exposure among pregnant women. Models 1 and 2 are summarized in Table 2. Model 1 was the base model in which only educational attainment, employment status, marital status and personal income per month were included, which showed that unemployed participants (OR = 1.658, 95% CI: 1.126–2.441) were more likely to be exposed to SHS at home. According to the Hosmer–Lemeshow test, we could see that Model 1 had a goodness-of-fit (χ^2^ = 4.288, *p* = 0.638). After controlling for covariates including age, ethnicity, household registration status, smoking habit, current exercising and drinking, employment status of spouses/partners, educational attainment of spouses/partners, drinking habits of spouses/partners in the last year, living with an aunt/uncle after getting married, regular antenatal examinations and complications of pregnancy, Model 2 showed that unemployed women (OR = 1.572, 95% CI: 1.013–2.441) had a higher risk for exposure to SHS than employed women. Participants with a high school or technical secondary school education were more likely to be exposed to SHS than women with a college education or above (OR = 1.601, 95% CI: 1.029–2.449). We could see that the fully adjusted binary logistic regression model had goodness-of-fit using the Hosmer–Lemeshow test (χ^2^ = 6.623, *p* = 0.578).

**Table 2:** Binary logistic regression models for the association between SES and SHS exposure among pregnant women in the 3rd trimester

	Model 1	OR(95% CI)	Model 2	OR(95% CI)
Marital Status				
Divorced but living together/Cohabiting relationship	0.537	0.232–1.244	0.586	0.237–1.448
Married and living together	1		1	
Employment				
No	**1.658**	**1.126–2.441**	**1.572**	**1.013–2.441**
Yes	1		1	
Educational attainment				
Junior middle school or below	1.161	0.739–1.824	1.362	0.825–2.249
High school or technical secondary school	1.317	0.879–1.972	**1.601**	**1.029–2.449**
College or above	1		1	
Personal income per month in RMB				
≤ 3000	0.759	0.392–1.469	0.869	0.426–1.771
3001–7999	0.864	0.576–1.295	0.934	0.604–1.446
≥ 8000	1		1	
Confounders				
Age	–		0.994	0.955–1.034
Household registration status (hukou)				
Rural area	–		1.014	0.670–1.533
Urban area	–		1	
Ethnicity				
Han	–		**4.983**	**1.027–24.177**
Minority group	–		1	
Smoking habit				
Never	–		1.558	0.627–3.870
Former	–		1	
Currently drinking				
Yes	–		1.616	0.907–2.880
No	–		1	
Currently exercising				
No			0.633	0.329–1.216
Yes			1	
Living with an aunt/uncle after getting married				
Yes	–		1.420	0.977–2.065
No	–		1	
Complications of pregnancy				
Yes	–		0.684	0.379–1.236
No	–		1	
Regular antenatal examinations				
No	–		0.457	0.207–1.010
Yes	–		1	
Employment of spouse/partner				
Unemployed	–		0.792	0.206–3.047
Employed	–		1	
Educational attainment of spouse/partner				
Junior middle school or below	–		0.625	0.369–1.057
High school or technical secondary school	–		0.729	0.481–1.103
College or above	–		1	
Drinking habit of spouse/partner in the last year				
No	–		**0.300**	**0.209–0.492**
Yes	–		1	
Cox & Snell R Square	0.020		0.131	
Nagelkerke R Square	0.027		0.181	

Values indicated in bold are statistically significant

Model 1 Includes marital status, employment status, educational attainment and personal income per month

Model 2 Further adjusted for age, ethnicity, household registration status, smoking habit, current drinking, current exercising, employment status of spouses/partners, educational attainment of spouses/partners, drinking habit of spouses/partners in the last year, living with an aunt/uncle after getting married, regular antenatal examinations and complications of pregnancy

### The interaction effect between SES and age on SHS exposure

Table 3 indicates the interaction effect between age and SES on SHS among pregnant women in the 3rd trimester. Herein, we observed that after controlling for covariates, significant interaction effects between marital status and educational attainment and age on SHS exposure were detected, with *P value*s for interaction of 0.009, 0.010 and 0.004, respectively.

**Table 3:** The interaction effect between age and SES on SHS exposure among pregnant women in the 3rd trimester

Model	Variables	Categories	*P* for multiplicative interaction
Model 1 ^**a**^	Marital status		
		Divorced but living together/Cohabiting relationship	**0.009**
		Married and living together	[reference]
Model 2 ^**b**^	Employment		
		No	0.950
		Yes	[reference]
Model 3 ^**c**^	Educational attainment		
		Junior middle school or below	**0.010**
		High school or technical secondary school	**0.004**
		College or above	[reference]
Model 4 ^**d**^	Personal income per month in RMB		
		≤3000	0.318
		3001–7999	0.313
		≥8000	[reference]

Values indicated in bold are statistically significant

^**a**^Model included marital status and marital status*age and was adjusted for employment, educational attainment, personal income per month, age, ethnicity, household registration status, smoking habit, currently exercising and drinking, employment status of spouses/partners, educational attainment of spouses/partners, drinking habit of spouses/partners in the last year, living with an aunt/uncle after getting married, regular antenatal examinations and complications of pregnancy

^**b**^Model included employment and employment*age and was adjusted for marital status, educational attainment, personal income per month, age, ethnicity, household registration status, smoking habit, current exercising and drinking, employment status of spouses/partners, educational attainment of spouses/partners, drinking habit of spouses/partners in the last year, living with an aunt/uncle after getting married, regular antenatal examinations and complications of pregnancy

^**c**^Model included educational attainment and educational attainment*age and was adjusted for marital status, employment status, personal income per month, age, ethnicity, household registration status, smoking habit, current exercising and drinking, employment status of spouses/partners, educational attainment of spouses/partners, drinking habit of spouses/partners in the last year, living with an aunt/uncle after getting married, regular antenatal examinations and complications of pregnancy

^**d**^Model included personal income per month and personal income per month *age and was adjusted for marital status, employment status, educational attainment, age, ethnicity, household registration status, smoking habit, current exercising and drinking, employment status of spouses/partners, educational attainment of spouses/partners, drinking habit of spouses/partners in the last year, living with an aunt/uncle after getting married, regular antenatal examinations and complications of pregnancy

Table 4 lists the results of the association between age and SHS exposure stratified by SES. We observed that age was a risk factor for SHS exposure in women who were divorced but living/cohabiting with a spouse/partner (OR = 1.256, 95% CI: 1.019–1.549). However, a decreased risk for exposure to SHS according to age was observed in participants with a college education or above (OR = 0.939, 95% CI: 0.884–0.997).

**Table 4:** Association between age and SHS exposure, stratified by SES

Model	Variables	Categories	OR(95% CI)	*P* for difference between stratums
Model 1 ^**a**^	Marital status			
		Married and living together	0.982 (0.943–1.023)	[reference]
		Divorced but living together/Cohabiting relationship	**1.256 (1.019–1.549)**	**< 0.05**
Model 2 ^**b**^	Educational attainment			
		Junior middle school or below	1.039 (0.945–1.143)	[reference]
		High school or technical secondary school	1.049 (0.969–1.135)	**< 0.05**
		College or above	**0.939 (0.884–0.997)**	**< 0.05**

Values indicated in bold are statistically significant

^**a**^Model 1 was adjusted for employment, educational attainment, personal income per month, ethnicity, household registration status, smoking habit, current exercising and drinking, employment status of spouses/partners, educational attainment of spouses/partners, drinking habit of spouses/partners in the last year, living with an aunt/uncle after getting married, regular antenatal examinations and complications of pregnancy

^**b**^Model 2 was adjusted for employment status, marital status, personal income per month, ethnicity, household registration status, smoking habit, current exercising and drinking, employment status of spouses/partners, educational attainment of spouses/partners, drinking habit of spouses/partners in the last year, living with an aunt/uncle after getting married, regular antenatal examinations and complications of pregnancy

## Discussion

To the best of our knowledge, there is a dearth of studies on the relationship between individual SES and SHS exposure at home among pregnant women in the 3rd trimester in China. This study not only provided insight into the status of SHS exposure but also examined its relationship with SES. Unemployment and high school or technical secondary school educational attainment had significant effects on SHS exposure. In the present study, we found that along with an increase in age, pregnant women with an unstable marriage (cohabiting relationship) and a college education or above were associated with an elevated and decreased risk of SHS exposure, respectively.

The finding showed that the prevalence of current SHS exposure in this study was 35.1%, which was lower than that of a previous national-level study from 2013 (47.2%) [2]. The prevalence of SHS exposure in this paper was also lower than that reported in prior population-based studies conducted in Henan Province and Sichuan Province [6, 7]. It could be perceived that the rate of SHS exposure among pregnant women varied by region, with some locations having a high level of exposure but other regions having a low level. In our study, we found that the prevalence of SHS exposure was higher in participants who lived with an aunt/uncle after getting married, which was consistent with a prior study [31]. This phenomenon might be explained by the high prevalence of smoking among middle-aged and older people in the central and western regions of China [43]. We also observed a high proportion of SHS exposure among participants who regularly received antenatal examinations (36.9%). This was because almost all of the pregnant women had received antenatal examinations and covered up the actual distribution of SHS exposure. Thus, expanding sample sizes and diversity is required to confirm the representativeness of our findings. As expected, we observed a higher proportion of SHS exposure in unemployed participants (44.7%), which revealed the potential influence of SES on SHS exposure. A previous study reported that pregnant women’s alcohol consumption increased the odds of environmental tobacco smoke (ETS) exposure during pregnancy [23] which was similar with our finding. However, the rate of SHS exposure was higher among participants who reported that their spouse/partner had no drinking habit in the last year than among those who reported that their spouse/partner drank in the last year. Currently, none of the prior literature demonstrated whether a spouse’s alcohol consumption could affect SHS exposure. This finding should not be over interpreted because investigation with large samples is warranted to show a more representative distribution among the population.

In summary, tobacco plays a very important role in China’s economy and culture. The government has tried to encourage adults to give up tobacco, but a large number of men still have smoking habits in China. Smoking is a widely accepted behavior in China, although SHS exposure has become a major public health problem and has caused a heavy burden of disease worldwide [44]. Thus, tobacco risks during pregnancy are common in the population of pregnant women, and some practical policies are urgently needed to protect their health.

Our results are in line with prior studies [23, 45] showing that women’s higher educational attainment was an independent protective factor for reducing SHS exposure. Education is one aspect of the basic drivers of human behavior that can promote healthy behavior and keep individuals from harmful exposure [46]. Generally, pregnant women who are more educated have greater awareness of tobacco and reduced exposure to smoke. Moreover, the availability of various medical and economic resources may depend on educational attainment. Thus, educational attainment can be regarded as a vital determinant of SHS exposure. In our study, we also confirmed that unemployed participants had a greater risk for SHS exposure. Employment status and education level are strongly associated, and both have impacts on household income and the social conditions of resources. Previous studies have shown that unemployment or manual labor are predictors of maternal SHS exposure during pregnancy [32, 47, 48]. Participants who were more educated were more likely to have steady jobs, which increased the likelihood of engaging in healthy behaviors and actively staying away from harmful exposures [49]. Conversely, unemployed women have limited health education resources, a low awareness of the harms of exposure to SHS and a self-perception of relatively low status within their family, increasing the possibility of SHS exposure. In summary, the significant results in our findings indicated that pregnant women that were less educated and unemployed might be more likely to exposure to SHS.

Notably, age influences the link between a higher level of educational attainment, unstable marital status and SHS exposure; that is, a lower level of SES increases the risk of exposure to SHS with increasing age in pregnant women. Currently, several studies have indicated that age is a potential factor of SHS exposure. Younger women were more likely to be exposed to SHS [10, 25, 50, 51], but in the study by St Helen et al., women aged over 35 years had higher levels of UC (urinary cotinine) due to SHS exposure at home [52]. However, the combined effects of age and individual SES on SHS exposure have not been studied previously. This modification effect is plausible because SES is a fundamental cause of health outcomes because it is closely associated with access to important resources and affects health through multiple mechanisms [53]. However, the capacity to use resources to gain a health advantage is increasingly weak in populations with relatively low SES with increasing age. In particular, women in families with traditional Chinese cultural backgrounds were expected to be obedient to their spouses. It could be speculated that with increasing age, pregnant women with a lower level of educational attainment and an unstable marriage were less likely to change the smoking behavior of their spouses/partners, and exposure to SHS might occur more frequently.

Avoiding SHS exposure during pregnancy is an important health priority for health care professionals and policymakers. However, researchers have stated that it is still difficult to eliminate SHS exposure during pregnancy in low- and middle-income countries (LMICs), including China. First, the awareness of harmful outcomes attributed to SHS exposure was lower in LMICs [54]. Second, pregnant women may not argue with men due to the existence of a male-dominated ideology, even though they realize the risk of SHS exposure [55]. Importantly, although smokers tried to avoid direct contact with pregnant women, SHS exposure was much more difficult to avoid. One important reason was that the hidden demon called “thirdhand smoke (THS)” still remained in the environment, especially on skin and clothes, which poses a new threat to pregnant women; nonetheless, pregnant women and their family members are rarely aware of the risk of THS exposure [56]. In addition, family consensus on smoking bans may be an effective strategy [31], as in pregnancy, women are well protected in the family, particularly by their spouses/partners, who value their advice. Hence, it is imperative for both pregnant women and their spouses/partners to be included in interventions for tobacco control and the ongoing implementation of SHS prevention and pregnancy health education.

Some limitations of this study should be recognized. First, the definition of SHS exposure was relatively limited in the present study; we only collected information on spouse/partner smoking status, given that pregnant women generally spend most of their time with their spouses/partners during pregnancy. However, the contribution of other sources of SHS exposure, such as workplaces and restaurants; and the smoking behavior data on others living in the home were unavailable. Moreover, the status of exposure to SHS was determined using participants’ self-reports, which might have led to a recall bias of the measurement effect of SHS exposure to some extent. However, prior investigations found that measuring SHS exposure by self-report is still a satisfactory and acceptable approach to determining SHS exposure and is widely used in an increasing number of studies [57]. This cross-sectional study had practical limitations in terms of causal inference. Longitudinal or qualitative research is needed to help identify the association between SHS exposure and SES and to interpret the effect of age over time. In addition, the SES of the spouses/partners also partially explained the association between SHS exposure and social status [27]. However, no significant differences were observed between these SES variables of the spouses/partners in terms of group comparison analysis. Last, the sample size we used is relatively small and is representative only for a context similar to China; the generalizability of our results should be confirmed in future studies.

In summary, risk perceptions and communication were related to SES [58], which could suggest that SES may have practical applications in reducing SHS exposure during pregnancy. Overall, this paper’s results indicated that, to a certain extent, a lower SES leads to a higher likelihood of SHS exposure. Thus, pregnant women with a low SES should be identified as a high-risk population, approaches that are helpful in eliminating SHS exposure should be implemented, and smoking behaviors of spouses/partners should be controlled.

## Conclusion

In conclusion, our findings showed that SHS exposure is common among pregnant women in the third trimester of pregnancy. SHS exposure is still a challenge for pregnant women’s health and is affected by education level and employment status. Namely, women with a high school or technical secondary school education had a higher risk of SHS exposure than those with a college education or above. Unemployed women are also identified as a high-risk population. The risk of SHS exposure showed increasing with age for pregnant women who have unstable marriages. However, pregnant women with a high education level could benefit from an older age, reducing the risk of SHS exposure. Thus, it is important to provide preventative strategies to reduce SHS exposure, especially for pregnant women with low SES and consider the effect of age on the SES-SHS association.

## Data Availability

Data are currently not available online. However, data can be made available to any interested person(s) by contacting the corresponding author via email.
